# Mismatch detection in DNA monolayers by atomic force microscopy and electrochemical impedance spectroscopy

**DOI:** 10.3762/bjnano.7.20

**Published:** 2016-02-09

**Authors:** Maryse D Nkoua Ngavouka, Pietro Capaldo, Elena Ambrosetti, Giacinto Scoles, Loredana Casalis, Pietro Parisse

**Affiliations:** 1 Elettra-Sincrotrone Trieste S.C.p.A., s.s. 14 km 163.5 in Area Science Park, Basovizza, Trieste, Italy; 2 INSTM – ST Unit, s.s. 14 km 163.5 in Area Science Park, Basovizza, Trieste, Italy; 3 University of Trieste, Via Valerio 9, Trieste, Italy; 4 Department of Medical and Biological Sciences, University of Udine, Udine, Italy

**Keywords:** atomic force microscopy, DNA monolayers, electrochemical impedance spectroscopy, hybridization, mismatches

## Abstract

**Background:** DNA hybridization is at the basis of most current technologies for genotyping and sequencing, due to the unique properties of DNA base-pairing that guarantee a high grade of selectivity. Nonetheless the presence of single base mismatches or not perfectly matched sequences can affect the response of the devices and the major challenge is, nowadays, to distinguish a mismatch of a single base and, at the same time, unequivocally differentiate devices read-out of fully and partially matching sequences.

**Results:** We present here two platforms based on different sensing strategies, to detect mismatched and/or perfectly matched complementary DNA strands hybridization into ssDNA oligonucleotide monolayers. The first platform exploits atomic force microscopy-based nanolithography to create ssDNA nano-arrays on gold surfaces. AFM topography measurements then monitor the variation of height of the nanostructures upon biorecognition and then follow annealing at different temperatures. This strategy allowed us to clearly detect the presence of mismatches. The second strategy exploits the change in capacitance at the interface between an ssDNA-functionalized gold electrode and the solution due to the hybridization process in a miniaturized electrochemical cell. Through electrochemical impedance spectroscopy measurements on extended ssDNA self-assembled monolayers we followed in real-time the variation of capacitance, being able to distinguish, through the difference in hybridization kinetics, not only the presence of single, double or triple mismatches in the complementary sequence, but also the position of the mismatched base pair with respect to the electrode surface.

**Conclusion:** We demonstrate here two platforms based on different sensing strategies as sensitive and selective tools to discriminate mismatches. Our assays are ready for parallelization and can be used in the detection and quantification of single nucleotide mismatches in microRNAs or in genomic DNA.

## Introduction

Most current technologies for genotyping and sequencing are based on DNA hybridization, exploiting the high grade of selectivity due to the unique properties of DNA base pairing. Although the understanding of the behaviour of nucleic acids on a solid surface has made huge progress from the seminal work of Southern [1] due to the rapid development of DNA microarray and DNA microarray-based techniques [2–3], there are still open questions and bottlenecks limiting the selectivity and the sensitivity of devices that are based on the hybridization of DNA [4]. One example is the detection of single nucleotide polymorphism (SNP) [5]. Single-base variations in a DNA/RNA sequence afflict 1 out of 1000 base pairs in the genome causing small differences in individuals belonging to the same species. This can lead to diseases [6–8] or drastically affect the response to pharmacological treatments [9]. SNPs are particularly relevant for applications in the field of pharmacogenomics and population genetics, as a diagnostic tool towards a personalized approach to diseases [10]. However, state-of-the-art devices still are not fully able to identify a single-base mismatch nor to unequivocally distinguish fully and partially matching sequences during hybridization [11–12].

The most common strategies for mismatch detection can be divided in three different categories: hybridization-based detection, detection based on thermal denaturation and protein-mediated detection [5]. For each strategy, different read-out systems and experimental designs have been reported, which include fluorescence [13], surface plasmon resonance [14–15], electrochemical [16–17], atomic force microscopy [18–19], colorimetric assays [20], Raman spectroscopy [21]. However, all these state-of-the-art technologies are limited in multiplexing implementation, mutation discrimination and/or sample throughput. Therefore the field is still open for an optimization of strategies to overcome the current limitations [22].

We present here two platforms, which are based on different sensing strategies, to detect mismatched and/or perfectly matched hybridization of complementary DNA strands into ssDNA oligonucleotide monolayers. The first platform exploits atomic force microscopy-based nanolithography (nanografting) to create ssDNA nano-arrays on gold surfaces and then AFM topography measurements to monitor the variation of the height of the nanostructures after loading the complementary/mismatched strands in the liquid cell. In the last years we optimized this nanomechanical approach, which is based in the different rigidity of ss- and dsDNA [23–25], enabling the ultrasensitive detection of biomarkers [26]. The second strategy exploits the change in capacitance during the hybridization process, measured at the interface between a ssDNA-functionalized gold electrode and the solution in an electrochemical cell. In a previous work we demonstrated the ability to follow the hybridization of perfectly matched sequences in real time through electrochemical impedance spectroscopy (EIS) measurements on extended ssDNA self-assembled monolayers (SAMs) [27]. Here we successfully tested EIS for the detection of mismatched sequences. From the analysis of hybridization kinetics we distinguished the presence of single or multiple mismatches and their relative position.

Both nanoarrays and EIS devices hold the premises for parallelization, multiplexing and low-volume analysis, making them amenable for point-of-care diagnostics of SNPs. Moreover a comparative analysis between the two techniques allows for a deep understanding of hybridization processes in the presence of single and multiple mismatches.

## Experimental

### Fabrication and measurement processes of AFM-based assays

Gold-coated substrates were immersed in 300 μM of top oligo(ethylene glycol)-terminated alkylthiols (TOEG6: HS–(CH_2_)_11_–(OCH_2_CH_2_)_6_–OH) ethanol solutions, overnight, to allow for the adsorption and assembly of a full monolayer with bio-repellent characteristics [28]. The samples were then removed from the solution, rinsed with ethanol and water to remove loosely bound molecules and placed in a customised liquid cell for the AFM experiments.

All AFM experiments were carried out on a XE-100 Park Instruments with a customised liquid cell. Si cantilevers (NSC36B Mikromasch, spring constant: 0.6 N/m) were used for the nanografting experiments. Briefly, the AFM tip is scanned at high load (approx. 100 nN) over the TOEG6 SAM, operating in a buffer solution (10 mM Tris-HCl, 1 mM EDTA, (hereafter TE), 1 M NaCl, pH 7.1) containing 5 μM thiolated ssDNA oligonucleotides. The applied load is sufficient to displace the TOEG6 molecules from the gold surface, which are subsequently locally substituted by the thiolated ssDNA molecules, creating ssDNA patches embedded in the surrounding TOEG6 carpet. Exchanging the buffer and the thiolated ssDNA probes, it is possible to sequentially immobilize different sequences on the same substrate. The parameters for nanografting have been properly chosen to obtain a surface density of probes optimal for the detection of target hybridization, following previous works of our group [23–25]. After the immobilization the ssDNA patches are measured through AFM topographic imaging in soft contact with standard silicon cantilevers (CSC38 Mikromasch, spring constant: 0.06 N/m) at 1 Hz scan rate, applying a force of 0.1 nN. Hybridization was monitored after the addition of the required target solutions (1 μM target in TE buffer 1 M NaCl) into the AFM liquid cell for 1 h. All DNA sequences used in the present work are listed in Table 1.

**Table 1:** List of the sequences used for the AFM and EIS experiments. The position of the mismatches are typeset in bold.

sequence name	sequence

HS-SNP-C	HS–(CH_2_)_6_–5’–tgataatcatta**c**aaaactgaaata–3’
HS-SNP-T	HS–(CH_2_)_6_–5’–tgataatcatta**t**aaaactgaaata–3’
SNP-coC	5’–tatttcagtttt**g**taatgattatca–3’
SNP-coT	5’–tatttcagtttt**a**taatgattatca–3’
HS_ssDNA	HS–(CH_2_)_6_–5’–caaaacagcagcaatccaaagatcagacacccgattacaaatgc–3’
cDNA_3MM	5’–**t**catttgtaatcgggtgtc**g**gatc**c**ttggattgctgctgttttg–3’
cDNA_PM	5’–gcatttgtaatcgggtgtctgatctttggattgctgctgttttg–3’
cDNA_2MM	5’–gcatttgtaatcgggtgtc**g**gatc**c**ttggattgctgctgttttg–3’
cDNA_DOWN	5’–tctttggattgctgctgttttg–3’
cDNA_UP	5’–gcatttgtaatcgggtgtctga–3’

### Fabrication and measurement processes for EIS-based assay

Detailed fabrication processes and layout of the electrochemical impedance spectroscopy experiments have been reported by Ianeselli and co-workers [27]. Briefly, the setup (a scheme is reported in Figure S1, Supporting Information File 1) consists of a glass slide with lithographically fabricated working (WE) and counter (CE) gold electrodes. The two electrodes are covered with insulating resist leaving exposed to the solution only the active part, to avoid spurious effects. To confine the drop of solution and to carefully position the reference electrode (a classical millimetre-sized Ag/AgCl pellet electrode) we placed around the electrodes a silicone circular cell (6 mm in diameter, 4 mm in height). The WE and CE electrodes were functionalized with thiolated ssDNA molecules using a well-established procedure for DNA SAMs on gold [23,29]. Initially the electrodes were wetted for 10 min with a drop of a high-ionic-strength buffer, TE 1 M NaCl, containing 1 μM thiolated ssDNA. In this way a low-density ssDNA SAM (about 2 × 10^12^ to 3 × 10^12^ molecules/cm^2^) was obtained [29]. After DNA-functionalization the devices were rinsed with the buffer solution used for the measurements, 100 mM KCl, and the capacitance at the electrode/electrolyte interface was measured. In the hybridization step the cell is filled with a drop of the same hybridizing buffer solution, 100 mM KCl, containing the complementary or partially complementary DNA strand at different concentrations.

The electrochemical current *I*_rms_ is monitored between WE and CE with a Heka PG340 USB potentiostat upon application of a 10 mV AC voltage at 100, 200, 250 and 400 Hz. In this regime of frequencies the total impedance is dominated by the capacitance at the electrode/electrolyte interface, allowing for the extraction of the differential capacitance simply from a linear fit of *I*_rms_. The functionalized electrodes can be regenerated after the hybridization process by means of a thermal treatment in TE buffer (pH 9) for 1 h in oven at a temperature 10 °C higher than the melting temperature of the used DNA sequence. The differential capacitance after the regeneration treatment maintains its original value within the error bars (Figure S2, Supporting Information File 1).

## Results and Discussion

### Atomic force microscopy-based assay

**Figure 1:**
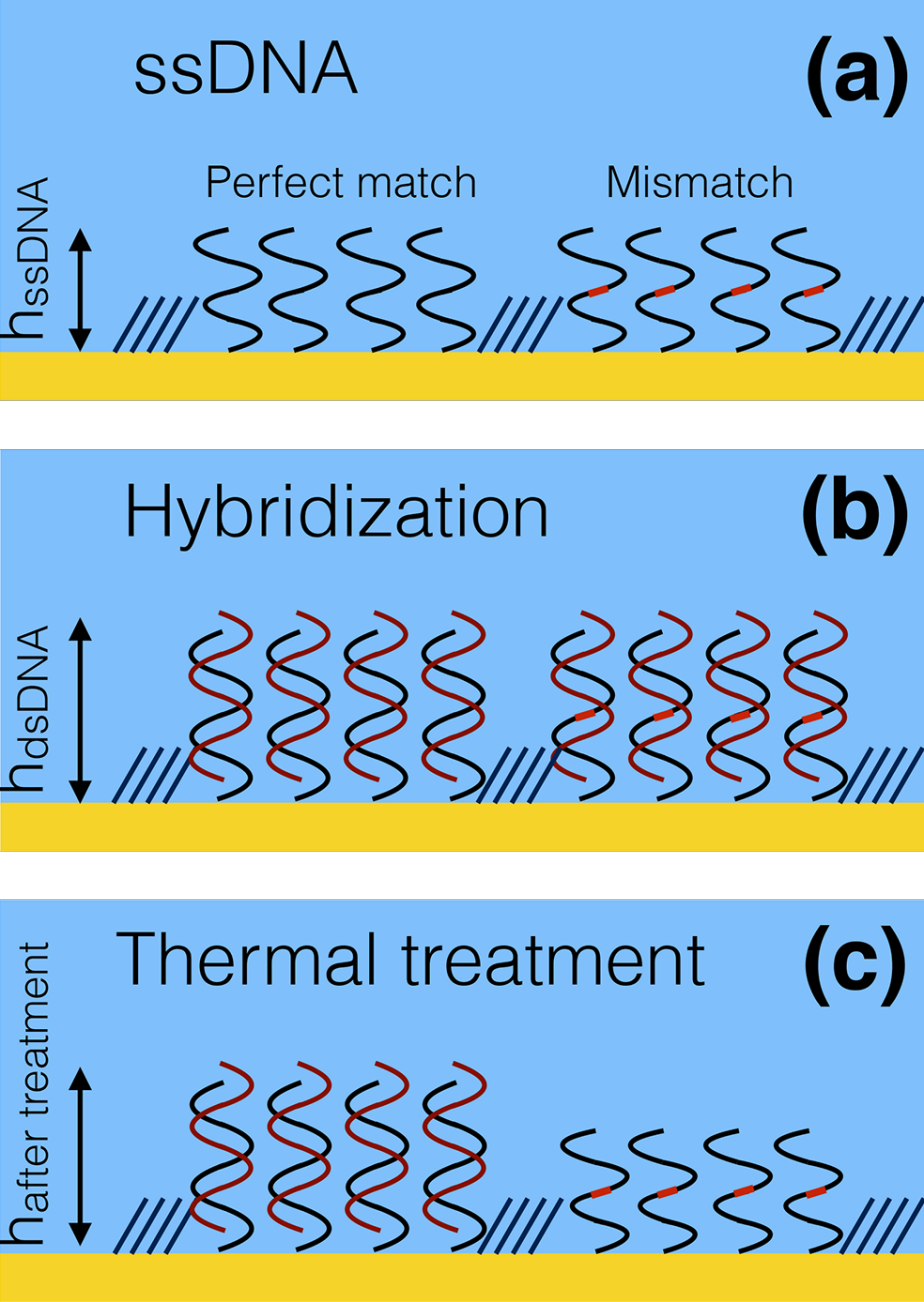
Schematics of the atomic force microscopy-based assay. We graft two sets of ssDNA nanostructures, whose sequences differ by one single base, highlighted by a red dot (panel a, b,c). By means of careful AFM topographic measurements, we record the height variation over the ssDNA nanostructures, (*h*_ssDNA_, panel a) upon hybridization with a strand fully matching only the left grafted strand (*h*_dsDNA_ panel b), upon thermal treatment, (*h*_after treatment_*,* panel c), evidencing the different de-hybridization behaviour of perfectly matched sequences vs mismatched sequences.

In Figure 1 we report a schematic representation of the AFM-based assay. We immobilize by means of nanografting on a gold surface two ssDNA sequences, differing by one base (reported as a red mark), and carefully measure the height of the DNA nanostructures with respect to the surrounding biorepellent self-assembled monolayer, this last serving as a constant reference for the height measurements (*h*_ssDNA_, Figure 1a). Then we hybridize with a sequence that is perfectly complementary to one of the two sequences. We expect the perfect matched (PM) sequence and the one-base mismatched (MM) sequence hybridization to produce a similar increase in height, which follows the change in the nanomechanical properties from ssDNA to dsDNA configuration (*h*_dsDNA_, Figure 1b). We then perform a thermal treatment to selectively de-hybridize only the MM sequences, as we can measure from the different height response of the two grafted ssDNA structures (*h*_after treatment_, Figure 1c). Since the non-perfectly matching sequence will have a reduced melting temperature with respect to the perfectly matched (PM) sequence (*T*_m_(MM) < *T*_m_(PM)), its de-hybridization will be favoured upon annealing to a temperature (*T*_ann_) close or slightly higher than the melting temperature of the perfect matched sequence (*T*_m_(MM) < *T*_m_(PM) ≤ *T*_ann_). We have used our AFM-based nanomechanical approach to distinguish single mismatched DNA base pairs of single nucleotide polymorphisms (SNPs), in particular a T–G mismatch.

**Figure 2:**
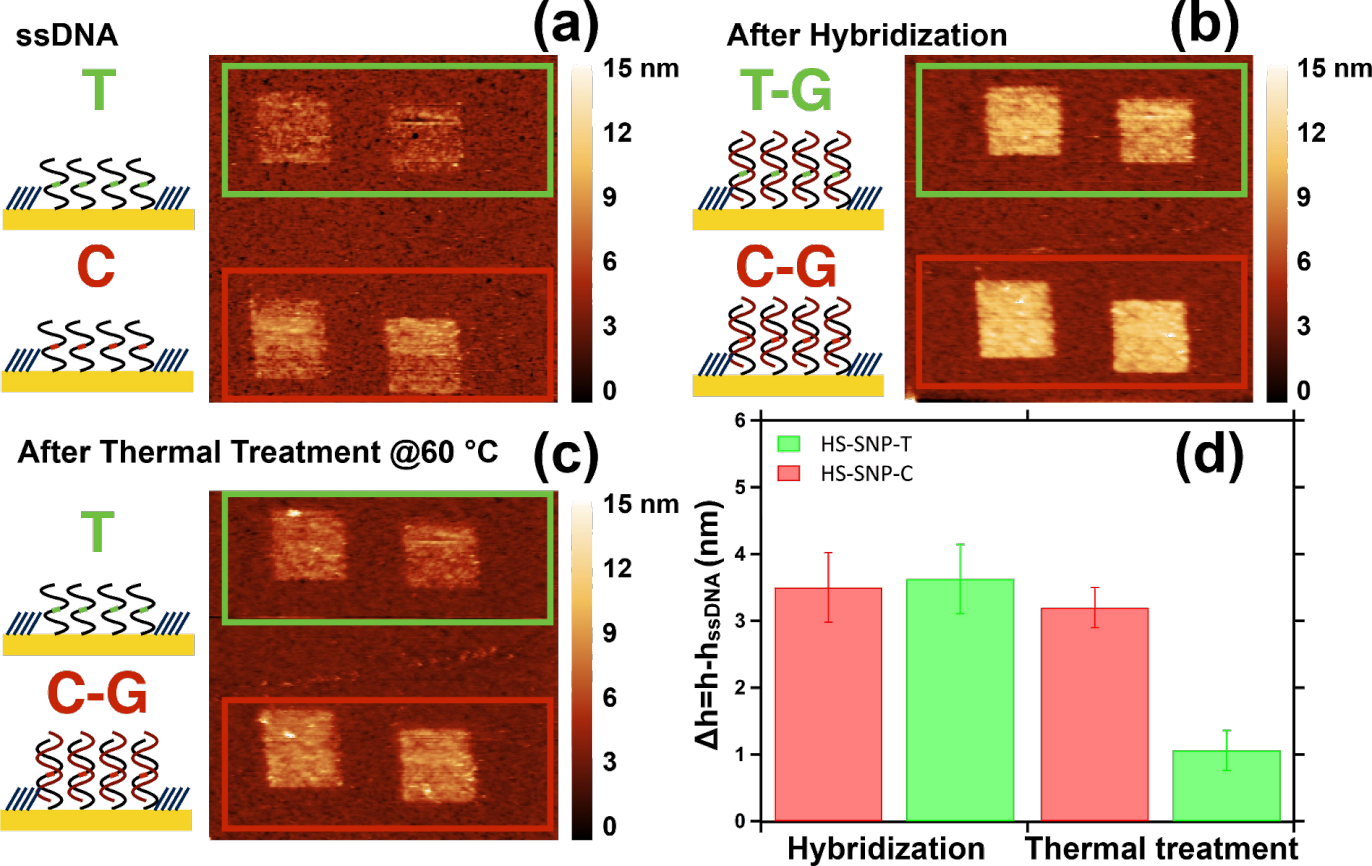
Schematic view and AFM topographic images of HS-SNP-C and HS-SNP-T nanografted patches (a) before and (b) after incubation with SNP-C for 1 h and (c) thermal treatment. (d) Histogram of the height variation with respect to the ssDNA patches (Δ*h* = *h* − *h*_ssDNA_) after the hybridization with SNP-C-Co sequence and after the thermal treatment (1 h at 60 °C).

In particular, we chose to immobilize on the surface two 25 bases-long ssDNA sequences, HS-SNP-C and HS-SNP-T (see Table 1) differing from one cytosine vs one thymine. We produced by nanografting patches of each of the two ssDNA sequences into 1 μm^2^ areas in the biorepellent TOEG6 SAM, using the same grafting parameters (Figure 2a). After grafting, the sample was incubated with the sequence SNP-coC fully matching one strand and matching the second one but for one base, originating a T/G polymorphism. In Figure 2b we report the AFM topographic image after incubation with SNP-coC targets for 1 h. The height variation (Δ*h* = *h* − *h*_ssDNA_) after the hybridization step is very similar for the two different sequences (Figure 2d), evidencing the impossibility to clearly distinguish the presence of the mismatched base only by means of height measurements. We therefore designed a melting experiment: we kept the sample in TE buffer, pH 9, for 1 h at 60 °C, a temperature slightly higher than the melting temperature of the PM sequence (*T*_m_^PM^ = 57 °C, *T*_m_^MM^ = 53 °C). In Figure 2c we report the AFM topographic image after the thermal treatment and in Figure 2d the relative height changes. We can observe a sensible height decrease in the HS-SNP-T probe only, matching almost completely the initial ssDNA value. This is the sign of a complete de-hybridization of the mismatched sequence, whereas the perfect match probe is only slightly perturbed by this thermal treatment.

These successful preliminary experiments demonstrate that our system has the ability to detect mismatches after precise annealing steps, as the ones used in current melting-based SNPs assays [30–31]. The novelty of our assay resides in the possibility of reducing the dimensions of the spots (below 1 μm^2^) and to work multiplexing in small volumes. The use of locked nucleic acids or enzyme-based strategies [22] might improve sensitivity further possibly circumventing the annealing step.

### Electrochemical impedance spectroscopy-based assay

**Figure 3:**
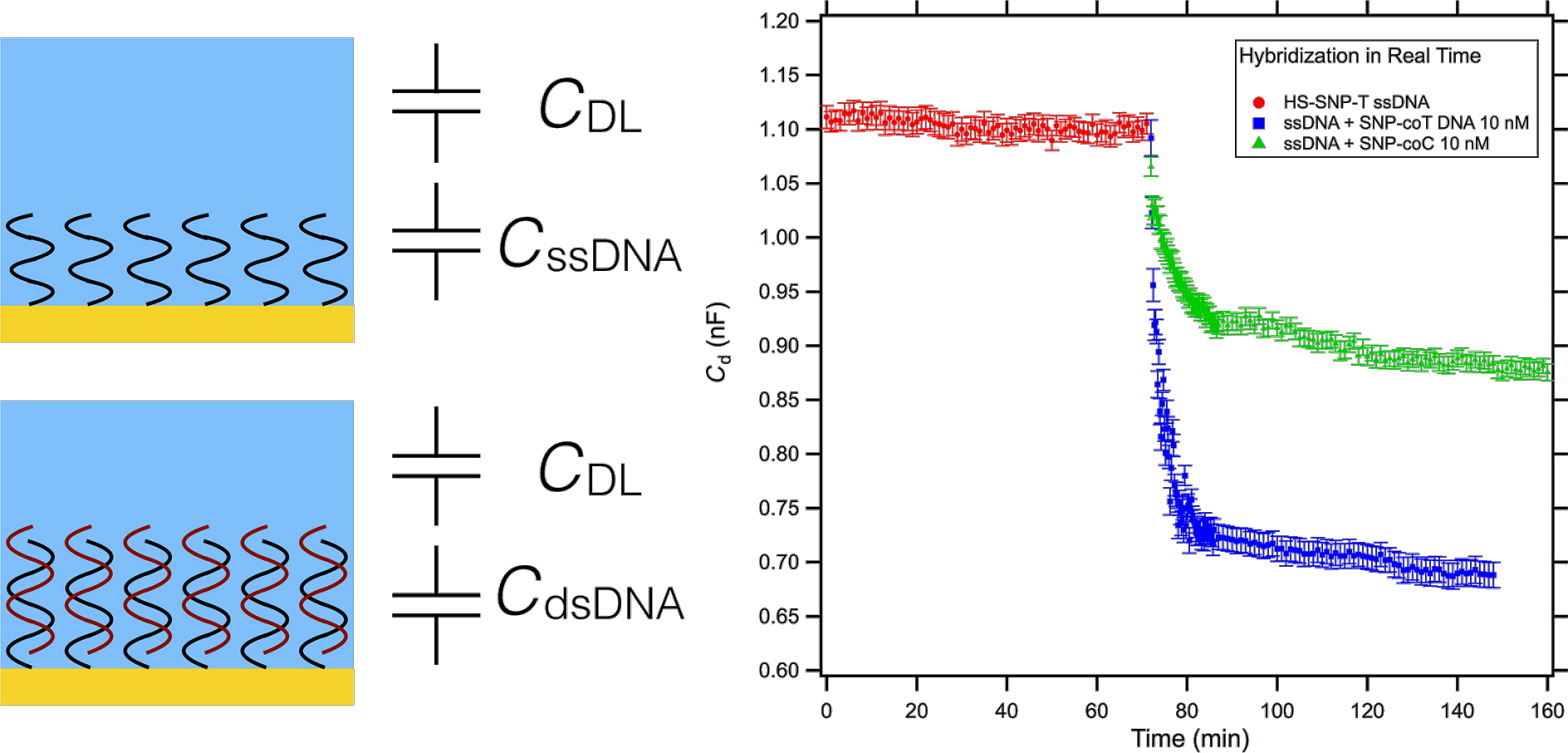
Schematic representation of the electrode/electrolyte interface. The first layer in contact with the gold electrode is the ssDNA self-assembled monolayer, modelled as a capacitance *C*_ssDNA_. Then we have the ions present in solution that arrange in response to the gold and DNA charges forming the so-called double layer capacitance *C*_DL_, in series with *C*_ssDNA_. When hybridization occurs, the binding of the complementary strand will produce a change in capacitance due to height changes, substitution of water molecules in the biological layer, and changes in the electrical charge density. The capacitance, extracted from the impedance measured in our electrochemical setup, is plotted versus time for the ssDNA-functionalized electrode (red curve) and for the mismatched (green) and perfectly matching (blue) complementary sequences.

Despite the high sensitivity, the AFM assay does not allow, at the moment, for a real-time investigation of binding events. In order to overcome such limitations, we tested in parallel another device developed in our laboratory [27], based on electrochemical impedance spectroscopy (EIS) [32]. The device exploits the capacitive effects at the interface between an electrode and an electrolytic solution. When a potential is applied to the gold electrode the free ions in solution will rearrange close to the surface creating the so-called double layer capacitance (*C*_DL_) [33–34]. In presence of a molecular layer between the solution and the electrode, an additional capacitance in series has to be taken in account. In our case, similarly to the AFM experiments, we functionalized the electrode with a low density ssDNA monolayer that serves as a probe for hybridization studies. In Figure 3 we report a scheme of the device as a series of two capacitances, one due to the charged DNA strands, *C*_ssDNA_ and the other (*C*_DL_) to the pure ionic solution [35]. The measurements of the total differential capacitance will be dominated by the smaller capacitance and, since *C*_ssDNA_ (densities of about 10 μF/cm^2^) < *C*_DL_ (densities of about 40 μF/cm^2^) [27], will give us a reasonable estimation of the *C*_ssDNA_. In the approximation of parallel plate capacitance we can write *C*_ssDNA_ as ε·ε_0_(*A*/*d*), where *A* is the area of the electrode, *d* the thickness of the ssDNA layer, and ε_0_ and ε are the dielectric constant of vacuum and ssDNA layer, respectively. When we insert a complementary strand in the electrochemical cell, the molecular recognition between the two strands will cause a change in the capacitance at the interface, due to a combination of height changes, displacement of water molecules upon binding of new strands, and rearrangement of charge density, bringing to a new value for the capacitance, *C*_dsDNA_ [36]. Our device can follow the variation of capacitance in real time, allowing for the study of the kinetic of hybridization. Indeed, the eventual presence of a mismatch should change the kinetic of the binding, as already reported by pioneering work of Georgiadis’s group [37–38]. Therefore, following in real time the variation of the capacitance we expect to distinguish the presence of mismatched sequences.

We functionalized the electrode with the HS-SNP-T probe and measured the capacitance at the electrode (red dots in Figure 3). The value of *C*_ssDNA_ is shown to be constant over an hour of continuous measurements, as already demonstrated by Ianeselli et al. [27]. After addition of the perfectly matching sequence SNP-coT (blue squares) in the electrochemical cell, we observed a fast decrease of the capacitance, followed by a subsequent slow decay that reaches a plateau at a value of capacitance 36% less than the initial value, as a sign of the occurred hybridization. When we insert on the regenerated electrode with the HS-SNP-T probe the mismatched sequence SNP-coC (green triangles) we observe a slower decay of the capacitance tending to a plateau much closer to the initial *C*_ssDNA_ value than the perfectly matched one (21% variation), confirming a different kinetic behaviour and a less efficient hybridization. Our results are in good agreement with previous reports of Georgiadis based on SPR measurements [38]. We can observe here that the EIS measurements allow for distinguishing the mismatched and perfectly matched sequences by observing a different kinetic behaviour and a different capacitance plateau, whereas AFM was not able to directly detect a height difference. Indeed, the changes of capacitance at the functionalized electrode are the results of a combination of changes of height in the molecular case and rearrangement of charge density. The distortions on the DNA structure due to the mismatched bases can modify the charge distribution inside the molecular layer [39], causing a change in the capacitance that is readable in the EIS measurement, even if does not significantly affect the height of the layer after the hybridization.

We further tested our device exposing a 44 bases ssDNA (HS_ssDNA_44) probe to five different sequences: a perfect match (cDNA_44_PM), a double mismatch (cDNA_44_2MM), a triple mismatch (cDNA_44_3MM), and two 22 bases sequences complementary to the bottom half (cDNA_44_DOWN) and top half (cDNA_44_UP) part of the ssDNA_44 sequence, respectively.

**Figure 4:**
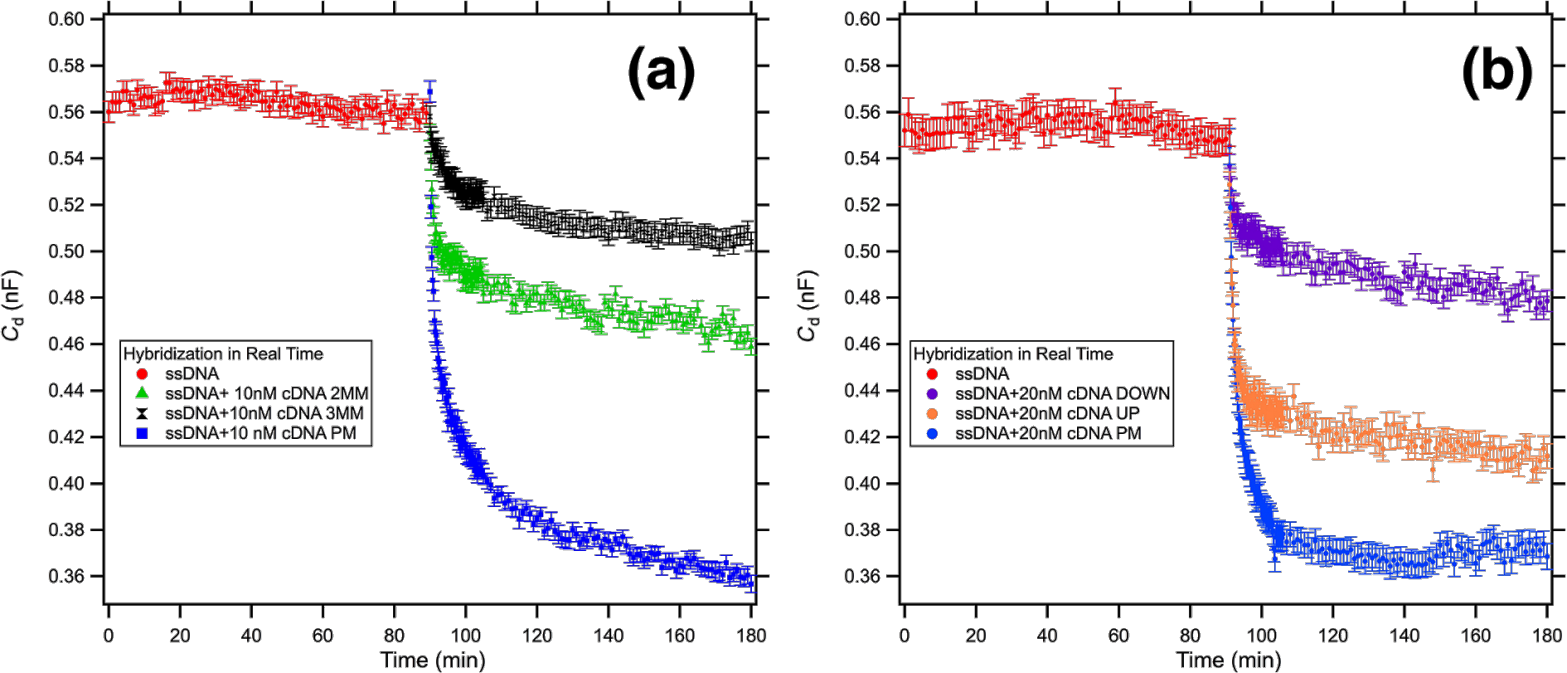
Differential capacitance measurements of the kinetics of DNA hybridization in presence of multiple mismatches (a) and in presence of partially complementary sequences (b). The red signal represents the differential capacitance of a low-density 44 bases ssDNA SAM functionalized WE measured in 100 mM KCl. (a) In blue we report the hybridization with the fully matching sequence, in green the hybridization with a sequence with 2 MMs, and in black the hybridization with a sequence with 3 MM. (b) In blue we report the hybridization with the fully matching sequence, in orange the hybridization with a 22mer sequence complementary with the upper part (far from the gold surface) of the target and in purple the hybridization with a 22mer sequence complementary with the lower part (close to the gold surface) of the target.

In Figure 4a we report the study of the kinetics of DNA hybridization in the presence of 2 MM (green triangles) and 3 MM (black markers) mismatches compared with the PM (blue squares) sequence. We can clearly distinguish the behaviour of the three differently matching sequences. As expected we measured a slower kinetics and a lower plateau value going from the PM (36% variation) to 2 MM (17% variation) and finally to 3 MM (10% variation). Analogously, we observe in Figure 4b the evolution of the differential capacitance in presence of two 22 bases-long sequences complementary to the bottom half (cDNA_44_DOWN) and top half (cDNA_44_UP) part of the ssDNA_44 sequence, respectively. The curves follow more or less the same trend: an initial fast decay and then a slow decay to an asymptotic value representative of the efficiency of the hybridization. The kinetics and the asymptotic value are, respectively, slower and lower for the two half sequences with respect to the PM. Notably, the kinetics and efficiency of hybridization is much lower for the down matching sequence than for the up matching sequence. The 44 bases probe brush can in fact hinder the hybridization of the bottom part, while the upper part is made more available for the target sequence. Noteworthy, we observe a sensible variation between up and down hybridization in the presence of as low as 20 nM target concentration. The increase in sensitivity with respect to previous results reported by Georgiadis group [38] can be attributed to the applied electric field during the EIS real time hybridization measurements. Such electric field can indeed favour the hybridization process, as already reported by [40], accelerating the kinetics and improving the efficiency of the hybridization.

## Conclusion

We proposed here two different sensing strategies based on the use of ssDNA monolayers tethered to gold substrates, for the detection of mismatches in DNA oligonucleotides. Both the strategies are label-free and are sensitive enough to detect point mutations. In Table 2 we report a comparison between the performance of our two approaches (nano-mechanical and electrochemical) and current label-free surface-based biosensing strategies, according to recent literature. As we can see from the table, SPR strategies seem to be the most promising in terms of limit of detection. However, in these devices the surface area is larger, limiting multiplexing and small volume operations [14–15].

**Table 2:** Comparison among different surface-based label free approaches for the detection of SNPs.

approach	detection limit	dimensions of the sensitive area	mutation discrimination	high throughput	multiplexing

AFM [11,18–19] and this work	100 pM to 1 μM	0.01–1 μm^2^	yes	not foreseen	yes
electrochemical [16–17] and this work	0.1 pM to 10 nM	10000 μm^2^	yes	yes, integrating with microfluidics	yes, integrating with microfluidics
surface plasmon resonance [14–15]	20 fM to 100 pM	more than 10000 μm^2^	yes	limited	limited

By contrast, the nano-mechanical approach on DNA nanoarrays although hampered by the time consuming processes of annealing and AFM height measurements (in line with benchmark of AFM-based assays reported in literature [11,18–19]), allows for a straightforward multiplexing. Ultimate sensitivity has been demonstrated for these arrays (100 pM, [41]), making them overall amenable to less invasive diagnostic analysis with a sensible reduction of the volume of the analyte till single cell [26].

Finally, our electrochemical measurements combine high sensitivity with real-time analysis, allowing for an accurate study of the kinetics and of the efficiency of the hybridization in mismatched targets. In our case, we were able to clearly distinguish the presence of single, or multiple mismatches and also the position with respect to the gold surface of the missing basepairs. Due to the relatively simple geometry, the device could be easily further miniaturized and integrated in multiplexed arrays through microfluidic systems, allowing for point-of-care diagnostics. Our results demonstrated that nano-mechanical and EIS strategies are state of the art for the detection of SNP, confirming the relevance of immobilized DNA on solid supports in life science studies, including single cell RNA characterization, gene expression profile and genetic variability. Moreover, the complementarity of the two techniques (one more sensitive to the morphological and mechanical changes of the DNA layer, the other more sensitive to its charge density) let us conclude that the structural deformations related to a single mismatch have a strong influence on the charge distribution only, leaving the molecular structure not significantly affected.

## Supporting Information

Supporting Information features a schematic view of the EIS setup, details of temperature stability of DNA nanobrushes for the AFM-based assays, and regeneration efficiency of ssDNA functionalized electrode for EIS measurements.

File 1 Additional experimental data.
