# Supplementary material for: Mismatch detection in DNA monolayers by atomic force microscopy and electrochemical impedance spectroscopy
Source: Beilstein J Nanotechnol. 2016 Feb 9;7:220–7. doi: 10.3762/bjnano.7.20 (PMC4778512; doi:10.3762/bjnano.7.20)
Supplement: File 1 — Additional experimental data. [file Beilstein_J_Nanotechnol-07-220-s001.pdf]

Supporting Information

for

Mismatch detection in DNA monolayers by atomic force microscopy and electrochemical impedance spectroscopy

Maryse D. Nkoua Ngavouka[‡1,2,3], Pietro Capaldo[‡1,3], Elena Ambrosetti[1,2,3], Giacinto Scoles[4], Loredana Casalis[1,2] and Pietro Parisse*[1,2]

Address: [1]Elettra-Sincrotrone Trieste S.C.p.A., s.s. 14 km 163.5 in Area Science Park, Basovizza, Trieste, Italy; [2]INSTM – ST Unit, s.s. 14 km 163.5 in Area Science Park, Basovizza, Trieste, Italy; [3]University of Trieste, Via Valerio 9, Trieste, Italy and [4]Department of Medical and Biological Sciences, University of Udine, Udine, Italy

Email: Pietro Parisse* - pietro.parisse@elettra.eu

* Corresponding author

‡ Equal contributors

Additional experimental data

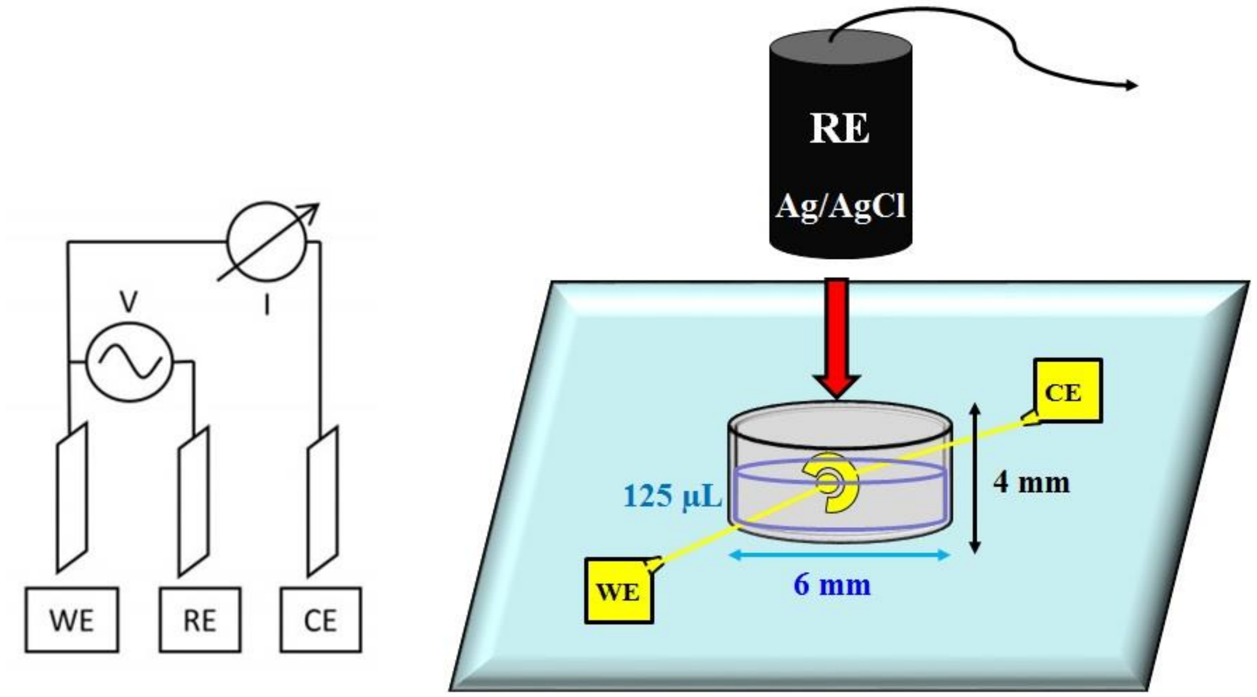

Figure S1: Schematic view of the electric scheme of the measurements and of the setup for the electrochemical impedance spectroscopy measurements. The working and counter electrodes, microfabricated on a glass slide, are immersed in silicon pool, filled with an ionic solution, with an inlet for the insertion of the standard Ag/AgCl reference electrode. An oscillating potential, referred to RE, is applied on the electrodes and the current flowing between the working and counter electrode is measured.

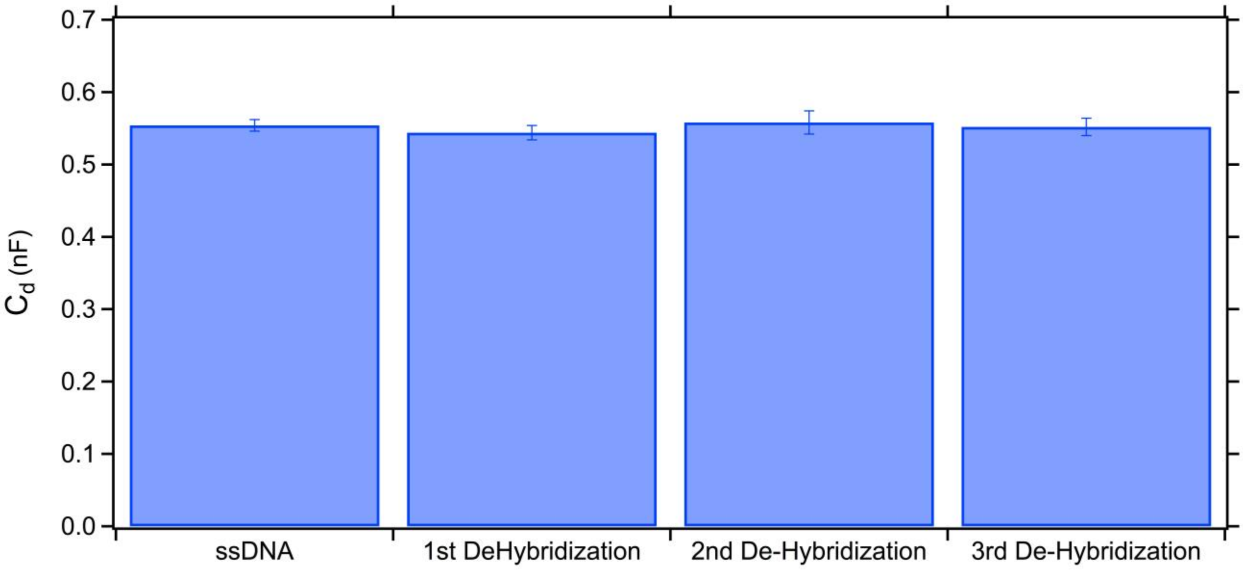

Figure S2: Values of differential capacitance averaged over one hour of measurements for an HS_ssDNA_44 probe functionalized electrode, immediately after the sample preparation (ssDNA) and after three different cycles of hybridization-dehybridization. For the regeneration we performed a thermal treatment in TE buffer (pH 9) for 1 h in oven at a temperature 10 °C higher than the melting temperature of the used DNA sequence). We can clearly observe that the functionalized electrode holds its differential capacitance value within the error bars after all the treatments.

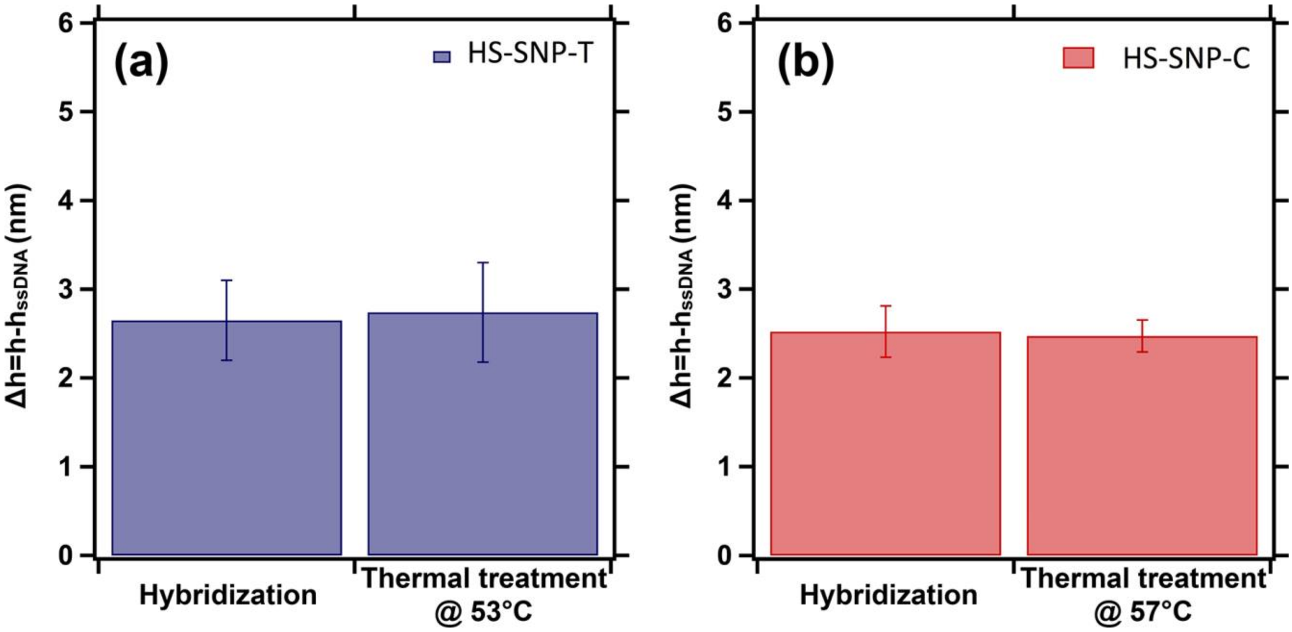

Figure S3: Height variation after hybridization of ssDNA nanobrushes with their respective perfectly matching (PM) targets (SNP-C and SNP-T) and thermal treatment at their respective melting temperatures (53 °C SNP-T and 57 °C SNP-C). The height increase after the hybridization is about the same for the two types of patches. Successively, we proceeded to anneal the samples at their respective melting temperatures (53 °C SNP-T and 57 °C SNP-C), for 30 min, in order to test the stability of the double strands. Working at the melting temperature should not separate all the hybridized strands. The height measured after annealing is almost unchanged for SNP-C and SNP-T. In any case, we can conclude here that 30 min the thermal treatment at the melting temperature is not enough to promote full dehybridization, in the case of fully matching sequences.
